# The variation of gut microbiota in captive Sichuan golden snub-nosed monkeys (*Rhinopithecus roxellana*) from infancy to adulthood

**DOI:** 10.3389/fvets.2025.1683047

**Published:** 2025-10-28

**Authors:** Rong Chen, Xinyi Liu, Siteng Wang, Lan Guo, Meirong Li, Xiaojuan Xu, Ran Lv, Litong Hong, Guodong Wang, Changlin Deng, Weidong Sun, Guangjin Liu

**Affiliations:** ^1^Department of Veterinary Hospital, Nanjing Hongshan Forest Zoo, Nanjing, China; ^2^Sanya Institute of Nanjing Agricultural University, Nanjing Agricultural University, Sanya, China; ^3^College of Veterinary Medicine, Nanjing Agricultural University, Nanjing, China; ^4^Key Laboratory of Animal Bacteriology, Ministry of Agriculture, Nanjing, China

**Keywords:** *Rhinopithecus roxellana*, captive, 16S rRNA high-throughput sequencing, gutmicrobiota diversity, potential pathogenic bacteria

## Abstract

*Rhinopithecus roxellana* (*R. roxellana*) is an endangered primate species, and its infant survival rate in captivity is extremely low. In this study, we conducted a comparative analysis of the gut microbiota from 8 infants (14–18 days old), 12 juveniles/subadults (2–5 years old), and 12 adults of *R. roxellana* (7–14 years old), which were kept at the Nanjing Hongshan Forest Zoo in Nanjing City, Jiangsu Province, China. Through the 16S rRNA high-throughput sequencing, we found the gut microbiota richness and beta diversity in captive infant *R. roxellana* were significantly lower than those in the non-infant groups. The relative abundance of Firmicutes positively correlated with increasing age, whereas the abundance of *Shigella/Escherichia* and *Akkermansia* was significantly higher in the infants and exhibited a decreasing trend with age. Meanwhile, several potential pathogenic bacteria, including *Clostridium perfringens*, *Staphylococcus aureus*, and *Shigella flexneri,* among others, were found to be abundant in the infant samples following the cultivable bacteria isolation. This research preliminarily investigated the gut microbiota development of captive *R. roxellana* and provided a valuable theoretical foundation for improving the healthy breeding of captive infant *R. roxellana.*

## 1 Introduction

The animal gut microbiota is a highly diversified ecosystem that assists its hosts in digesting plant polysaccharides and fibers, thereby enhancing the animals’ ability to obtain nutrients from food (1). Meanwhile, the gut microbiota can prevent the colonization of pathogens in the host’s gastrointestinal tract, which is crucial for maintaining the balance of the mucosal environment, gut maturation, and gut integrity (2–4). The intestinal microbiota is susceptible to changes in response to alterations in feeding habits throughout the organism’s development (5–8).

The Sichuan golden snub-nosed monkey (*Rhinopithecus roxellana*, *R. roxellana*) is classified as an endangered primate by the International Union for Conservation of Nature (IUCN) (9). It is found only in alpine forests at altitudes ranging from 1,500 to 3,300 meters in China (10). Owing to habitat loss and fragmentation, captive breeding is crucial for enhancing the reproductive success of *R. roxellana* and aiding in the recovery of its population (11). In captivity, *R. roxellana* primarily consumes breast milk during infancy (0–6 months), while subadult (1.5–6 years) and adult (7–13 years) feed on leaves from various plants, fruits, vegetables, grains, and protein sources. This feeding change occurs alongside the development of their intestines and stomach. However, based on our clinical experience, the timid infants of *R. roxellana* are difficult to raise in captivity and prone to symptoms such as vomiting, diarrhea, and constipation. The survival rate of captive *R. roxellana* is therefore extremely low, with a high mortality rate (44%) during the infant and sub-adult stages (12).

The gastrointestinal disorders in captive *R. roxellana* predominantly occur during the food conversion period, which spans from 3 to 6 months of age, also known as the weaning period. We speculated that various gastrointestinal diseases could be associated with an imbalance in the gut microbiota. The diet structure of infant *R. roxellana* changes from breast milk to a vegetarian diet during the weaning period. The change in diet affects the composition of the intestinal microbiota and leads to disrupt the microbes balance, so that the potential pathogenic bacteria could colonize in the intestines of infant *R. roxellana* and cause diseases. However, few studies have addressed the impact of feeding changes on the gut microbes of infant *R. roxellana*. Therefore, comparing the gut microbiota characteristics of this primate at infant and non-infant stages, and particularly characterizing gut microbiota diversity in infants, may guide husbandry changes that could improve the survival of infant *R. roxellana* in captive breeding.

Although high-throughput sequencing technology has been used to explore the gut microbiota community of *R. roxellana* (13–15), the characteristics of *the* gut microbiota in infant *R. roxellana* remain unknown. In this study, the gut microbiota characteristics of 8 infant and 24 non-infant monkeys were compared using 16S rRNA high-throughput sequencing of noninvasive sampled feces, from which cultivable bacteria were also obtained. The purpose of this study is to better understand the changing characteristics of the intestinal microbiota in *R. roxellana* when their diets transition from breast milk to leaf-based.

## 2 Materials and methods

### 2.1 Ethical approval

All applicable international, national, and institutional guidelines for the care and use of animals were strictly adhered to in our research. All research reported in this study complied with the protocols approved by the Experimental Animal Welfare and Ethics Committee of Nanjing Hongshan Forest Zoo in Nanjing, China. The ethics certificate number is NHFZ2021030401, and the research followed the American Society of Primatologists (ASP) Principles for the Ethical Treatment of Non-Human Primates, as well as the ASP Code of Best Practices for Field Primatology, and conformed to the legal requirements of China.

### 2.2 Sample collection

The *R. roxellana* captive population is kept at the Nanjing Hongshan Forest Zoo (32°5′38.4″N, 118°48′2.48″E) in Nanjing City, Jiangsu Province, China, and is naturally divided into two groups that share the same outdoor playground. Nanjing features a subtropical monsoon climate, with an average elevation of around 20 meters above sea level. The climate is mild and has distinct seasons, creating a favorable environment for the captive population of *R. roxellana*. The sole food source for infant *R. roxellana* is its mother’s milk (Supplementary Figure S1). For non-infant *R. roxellana*, zookeepers provide approximately 1,350–1,600 grams of vegetables (including lettuces, carrots, and eggplants), 200 grams of fruits (such as apples, papayas, and oranges), 120–170 grams of macronutrient rich foods (sources including wheat, soybean meal, maize, and milk), and a sufficient amount of leaves from mulberry, maidenhair, acacia, and elm trees every day.

We divided the 32 healthy *R. roxellana* individuals, which showed no symptoms of illness, into three groups based on their age, in accordance with the *R. roxellana* husbandry guidelines (11): infants (1 month; females, *n* = 4; males, *n* = 4), juveniles/subadults (1.5–6 years; females, *n* = 7; males, *n* = 5), and adults (7–12 years; females, *n* = 7; males, *n* = 5). The detailed age and sampling time information were shown in Supplementary Table S1. The number of infants was small compared to the non-infants, mainly because only eight infants were born in the year we conducted our sampling, and this sample size for infants in the captive *R. roxellana* population has been quite substantial.

Since *R. roxellana* is a seasonal breeder and the infants are usually born from March to April, our sampling period for infant fecal samples in this study was in April. During this time, fresh feces from non-infant *R. roxellana* were also collected. Fresh fecal samples were collected immediately after defecation. Sterile gloves and bamboo sticks were used to remove the outer layer of the feces, and the inner section was sampled into two sterile centrifuge tubes. One tube was immediately frozen in liquid nitrogen and stored at −80 °C until DNA extraction; the other was transported immediately onto sheep blood agar plates for bacterial culture.

### 2.3 16S rRNA gene amplification and sequencing

Total DNA from fecal samples was extracted using the OMEGA DNA kit (M5635-02) (OMEGA Bio-Tek, Norcross, GA, United States). The quantity and quality of the extracted DNA were measured using a NanoDrop NC2000 spectrophotometer (Thermo Fisher Scientific, Waltham, MA, United States) and agarose gel electrophoresis.

The V3-V4 region of the bacterial 16S rRNA gene was selected as the target sequence and polymerase chain reaction (PCR) were performed with forward primer 338F (5′-ACTCCTACGGGGAGGAGCA-3′) and reverse primer 806R (5′-GGACTACHVGGGTWTCTAAT-3′). Each PCR reaction contained 12.5 μL of Taq Mix, 1 μL of DNA template, 1 μL of each upper and lower primer, and 9.5 μL of double-distilled water. Thermal cycling conditions were as follows: denaturation at 98 °C for 5 min; 25 times denaturation at 98 °C for 30 s, annealing at 50 °C for 30 s, extension at 72 °C for 45 s; extension at 72 °C for 5 min, then hold at 4 °C. We purified the PCR products using Vazyme VAHTS DNA cleaning beads (Vazyme, Nanjing, China) and quantified them with Quant-iT PicoGreen double-stranded DNA detection kit (Invitrogen, Carlsbad, CA, United States). The concentrations of the purified DNA ranged from 4.45 to 28.04 ng/μl, which is appropriate for sequencing. Sequencing was conducted using the Illumina MiSeq platform and the MiSeq kit v3 at Paisenor Biotech in Shanghai, China.

### 2.4 Biostatistical analysis

We performed microbiome bioinformatics analysis using QIIME2 (16, 17). The Cutadapt plugin (18) was used to remove the primer sequences from the raw sequence data, demultiplex the raw sequences using demux software, and assigned them to each sequence based on their bar codes. The sequences underwent quality filtering, denoising, merging, and chimera removal using the DADA2 plugin (19). After quality-control by DADA2, we aligned the sequences, termed non-monomeric amplicon sequence variants (ASVs) with MAFFT (20), and reconstructed their phylogenetic relationships with FastTree2 (21).

QIIME2 was utilized to assess the sequencing depth and to generate sparse curves from the sequencing data. We utilized R software (22, 23) to generate rank-abundance curves, which enabled us to compare the abundance and evenness of ASVs across each sample, as well as the log2-transformed ASV abundance. Sparsity curves and species accumulation curves were utilized to evaluate the sequencing depth. Alpha diversity, which refers to the diversity within a specific environment or ecosystem, is also known as within-habitat diversity (24). The Shannon diversity index (Shannon) reflects species richness and evenness, and is used to describe the distribution of species within the microbiota. The magnitude of this index indicates the degree of imbalance in the distribution (25), as calculated with QIIME2. Beta diversity reflects differences in microbial communities between groups, therefore, ASVs were classified first using the classify-sklearn naive Bayes classifier (26), followed by principal coordinate analysis (PCoA). We then employed the unweighted pair group method with arithmetic means (UPGMA) (17, 27) to compare sample microbial compositions and identify differences between age groups. Linear discriminant analysis effect size (LEfSe) (28) was performed to identify the taxa that differed among three age groups. PICRUSt2 is used to cluster ASV information in comparison with the sequenced microbial genome database, and subsequently, the functional types and abundances of the corresponding species are obtained from the MetaCyc Metabolic Pathway Database.

The Kruskal-Wallis test (29) was used to compare the alpha diversity and the relative abundance of bacteria at the phylum and genus levels across the three age groups. We calculated the median and interquartile range (IQR) using the Row statistics feature in GraphPad Prism version 9.0.0 (30). We utilized Dunn’s tests (31) to compare groups two by two when their comparisons exhibited statistically significant differences. We employed Permutational Multivariate Analysis of Variance (PERMANOVA) to assess unweighted UniFrac distances in beta diversity (32). For all tests, we set *a p*-value of less than 0.05 as our threshold for statistical significance.

### 2.5 Bacterial isolation and identification

Each fresh fecal sample was soaked in 1,000 μL PBS for 30 min and then 100 μL supernatant was coated directly onto 8% sheep blood agar plates. The plates were then incubated in an anaerobic incubator at 37 °C for 24 h, after which the colonies with different phenotypes were isolated and purified. Upon confirming that the clone was a pure bacterium through Gram staining, total DNA was extracted from the clone and subjected to PCR amplification using universal primers for the bacterial 16S rRNA gene (upstream primer: 5′-AGAGTTTGATCCTGGCTCAG-3′, downstream primer: 5′-GGTTACCTTGTTACGACTT-3′, synthesized by Nanjing Kingsray Biologicals). The amplification system with total volume 50 μL containing 25 μL of 2 × Taq PCR Master Mix (Nanjing Novozymes), 21 μL of dd H_2_O, 1 μL of each upstream and downstream primers, 2 μL of DNA template. Thermal cycling consisted of initial denaturation at 94 °C for 5 min, followed by 30 cycles consisting of 94 °C for 30 s, 55 °C for 45 s, 72 °C for 90 s, with final extension for 10 min at 72 °C. We used dd H_2_O as a negative control. The positive PCR products, which exhibited a band of approximately 1,500 bp on 1% agarose gel electrophoresis, were purified and sent for sequencing (Nanjing DynaScience Biotechnology). The sequencing results were submitted to NCBI BLAST to identify the bacteria: sequences of >95% identity represent the same genus, whereas sequences of >97% identity represent the same species (33).

## 3 Results

### 3.1 Sequence and data analysis

High-throughput sequencing of 32 fecal samples yielded 3,040,130 raw reads, with an average of 95,004 sequences per sample (ranging from 73,827 to 108,407). A total of 1,927,800 valid reads (with a mean of 60,243 sequences across samples, ranging from 32,439 to 87,902) were obtained after Quality filtering, chimera elimination, and duplicate data elimination. The accession number for each sequencing sample was list in Supplementary Table S1. The sparsity curves converged asymptotically, indicating that the reads were sequenced deeply enough to cover most of the 16S rRNA gene sequences in the 32 samples (Supplementary Figure S2a). Species rarefaction curves approached a plateau, reflecting the homogeneity and richness of each sample (Supplementary Figure S2b).

### 3.2 Alpha diversity of gut microbiota

**Figure 1:**
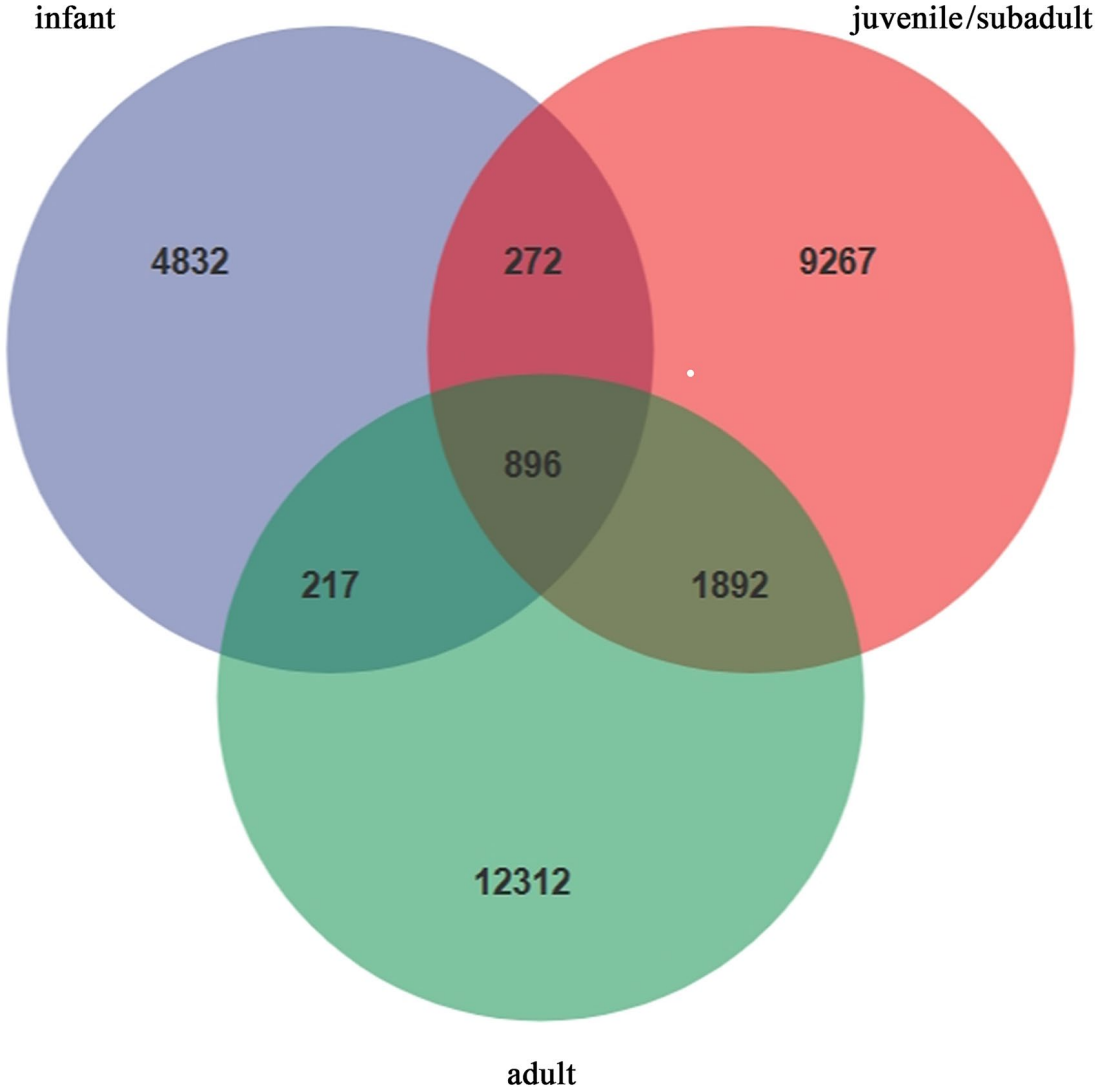
Wayne’s diagram of ASVs of gut microorganisms in *R. roxellana* at different ages.

The infant group had 6,217 ASVs, the juvenile/subadult group had 12,327 ASVs, and the adult group had 15,317 ASVs, as shown in Figure 1. There were 896 core ASVs across all samples within the three age groups, while 4,832 ASVs were unique to infant *R. roxellanae*. As evidenced by the distribution of identical ASVs, the juvenile/subadult group shared more ASVs with the adult group than with the infant group (Figure 1).

**Figure 2:**
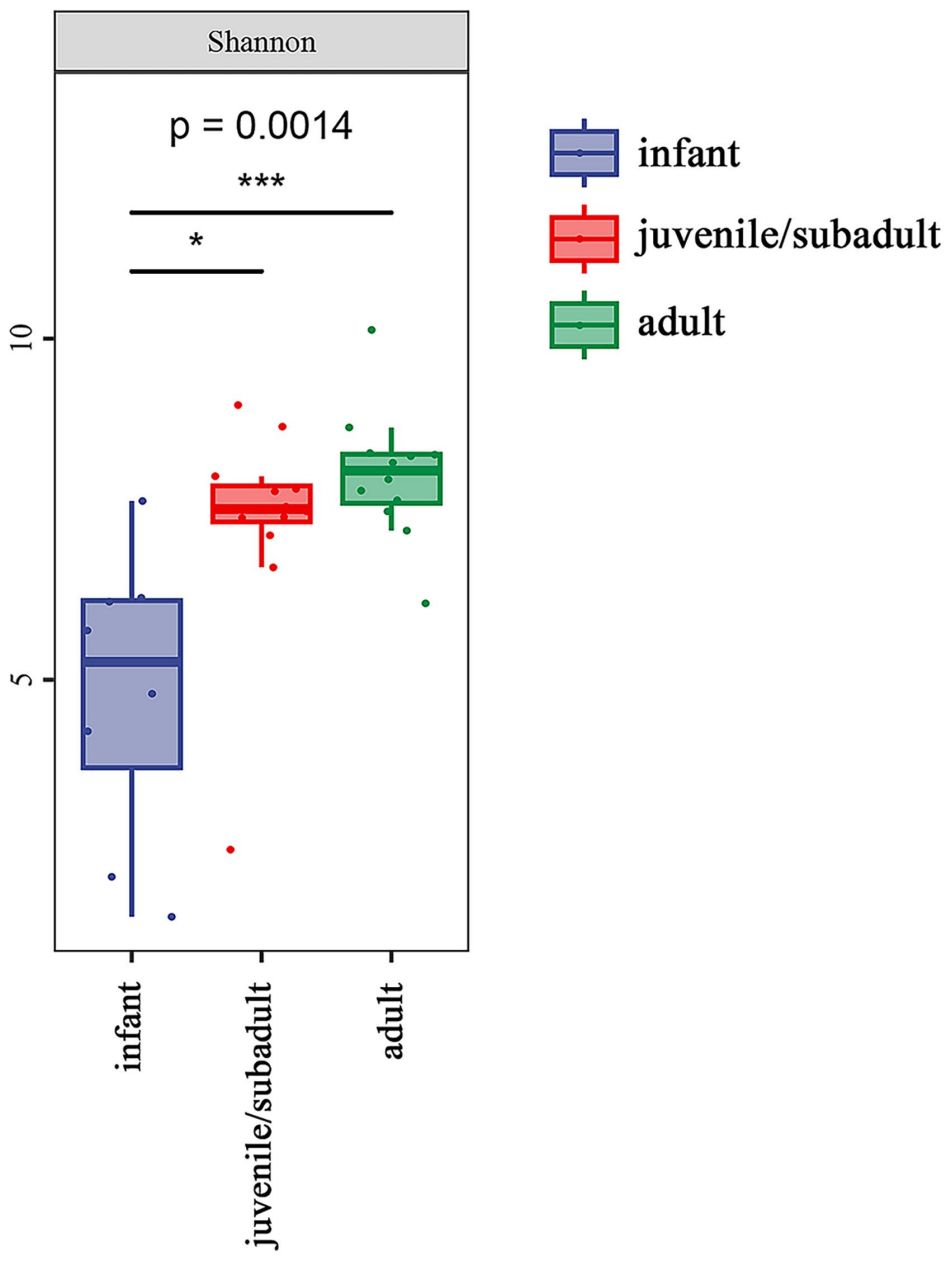
Alpha diversity of the gut microbiota in *R. roxellana* at Nanjing Hongshan Forest Zoo, China, March 2022: alpha diversity as measured by the Shannon index (Kruska-Wallis chi-squared = 13.92, df = 2, *p* = 0.0014). * indicates *p* < 0.05, *** indicates *p* < 0.001.

Alpha diversity analysis was measured using the Shannon index, which was 4.798 for infants, 7.277 for juveniles/subadults, and 8.003 for adults. The alpha diversity of the gut microbiota significantly differed among age groups (Figure 2), particularly between the infant and adult groups.

### 3.3 Beta diversity of gut microbiota

**Figure 3:**
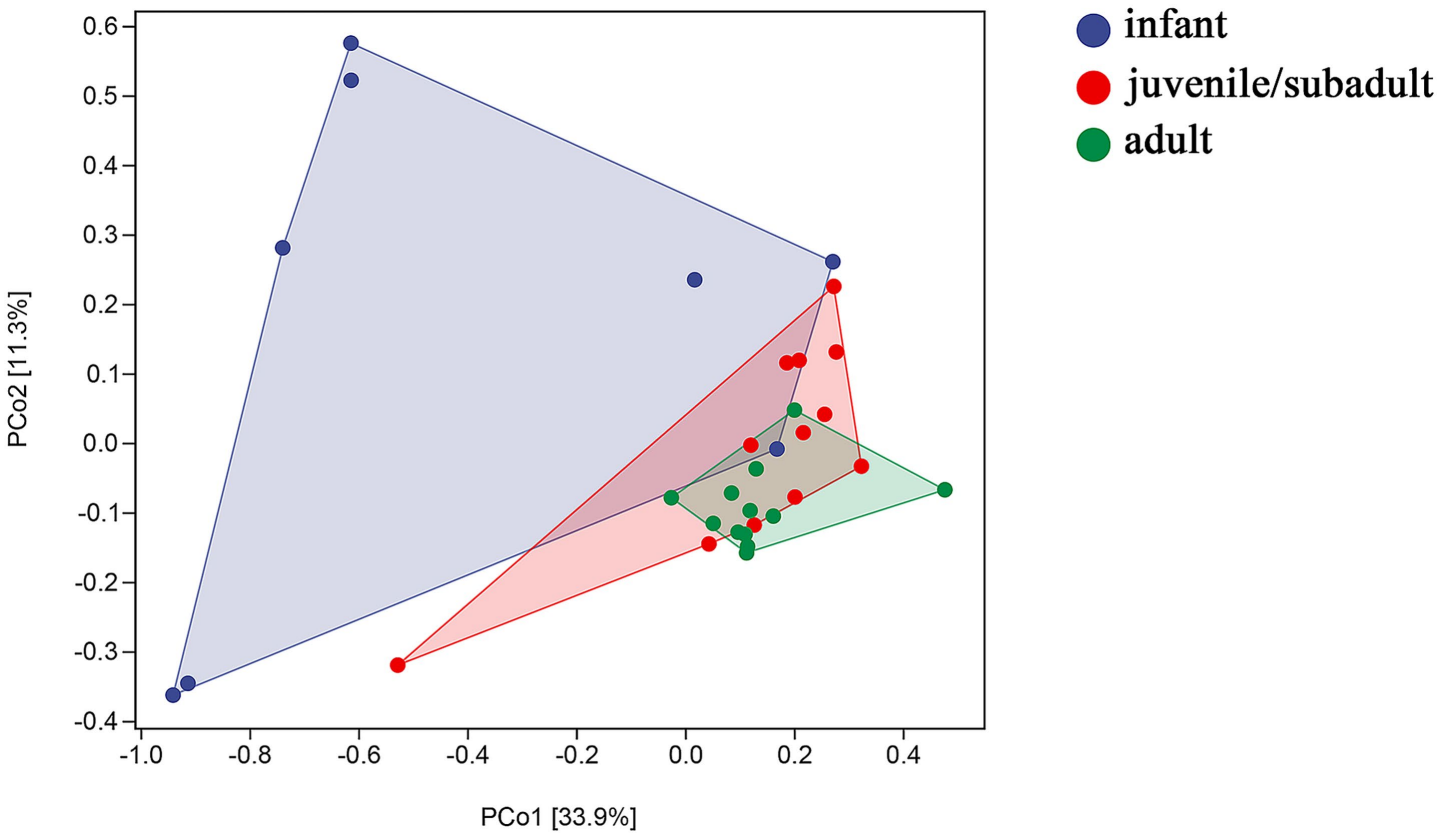
Weighted UniFrac distance of gut microbiota by PERMANOVA.

Principal coordinates analysis (PCoA) revealed differences in beta diversity among the gut microbiomes of three age groups. In Figure 3, PERMANOVA indicated that the sample distribution of the infant group was more dispersed, and the infant group showed significant differentiation compared to both the juvenile/subadult group (*p* = 0.003, df = 20) and the adult group (*p* = 0.001, df = 20). However, the sample distributions of the juvenile/subadult and adult groups could be clustered into one group (*p* = 0.077, df = 24). This suggests that significant gut microbial succession occurs from the infant to the subadult group.

### 3.4 Comparative analysis of the gut microbiota composition of *R. roxellana* of different ages

**Figure 4:**
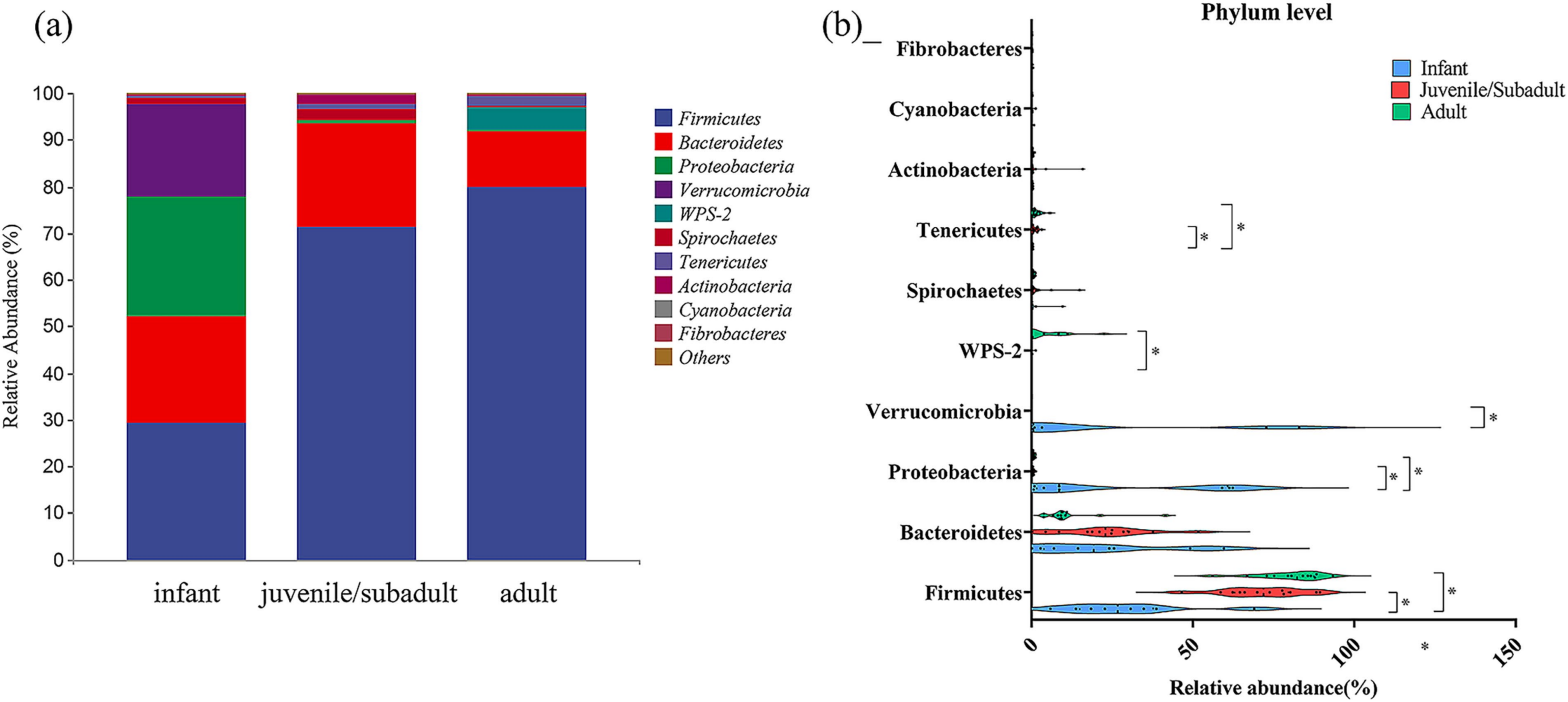
Relative abundance of gut bacteria at Phylum level in captive *R. roxellana* at Nanjing Hongshan Forest Zoo, China, March 2022. **(a)** In each age-group; **(b)** comparison of the relative abundance at Phylum level in different age classes. The violin plot range indicates the density of the distribution of the data. The straight line represents the median and dots indicate the individual datapoints. * indicates *p* < 0.05, *** indicates *p* < 0.001.

Based on the taxonomic annotation, all ASVs from each sample comprised 30 phyla (29 bacterial and 1 archaeal) (Figure 4a). At the phylum level, Firmicutes (29.25%), Bacteroidetes (22.82%), Proteobacteria (25.64%), and Verrucomicrobia (20.01%) were the predominant phyla in the intestinal microbiota of infants. Firmicutes (71.26%), Bacteroidetes (22.31%), Spirochaetes (2.21%), Actinobacteria (1.95%), and Tenericutes (1.11%) were the dominant phyla in the juvenile/subadult group. In the adult group, Firmicutes (79.70%), Bacteroidetes (11.93%), WPS-2 (4.53%), and Tenericutes (1.18%) were the major phyla.

Firmicutes, as one of the dominant phyla, contributed a relative abundance with a median of 26.706% (IQR: 5.866–68.986%) in infants, a median of 71.8401% (IQR: 46.5003–89.391%) in juveniles/subadults, and a median of 82.289% (IQR: 56.049–93.3619%) in adults (Figure 4b). Thus, Firmicutes differed significantly from infants to non-infants (Kruskal-Wallis test, H = 16.76, *p* = 0.0002, df = 2). Detailed results are available in Supplementary Table S2.

The relative abundance of Proteobacteria in the infant intestinal microbiota [median 8.582%, IQR (0.646–62.321%)] was significantly higher (Kruskal-Wallis test, H = 14.82, *p* = 0.0006, df = 2) than that of juveniles/subadults [median 0.4202%, IQR (0.162–1.274%)] and adults [median 0.495%, IQR (0.252–1.235%)]. Verrucomicrobia was also among the phyla with a high relative abundance in the gut during infancy [median 0.561%, IQR (0.004–82.947%)], after which the percentage declined and differed significantly from that of juveniles/subadults (Kruskal-Wallis test, H = 8.956, *p* = 0.0114, df = 2). The relative abundance of WPS-2 and Tenericutes increased with age, while infantile WPS-2 was significantly lower than that of adults, and Tenericutes in infancy were significantly lower than in non-infancy.

**Figure 5:**
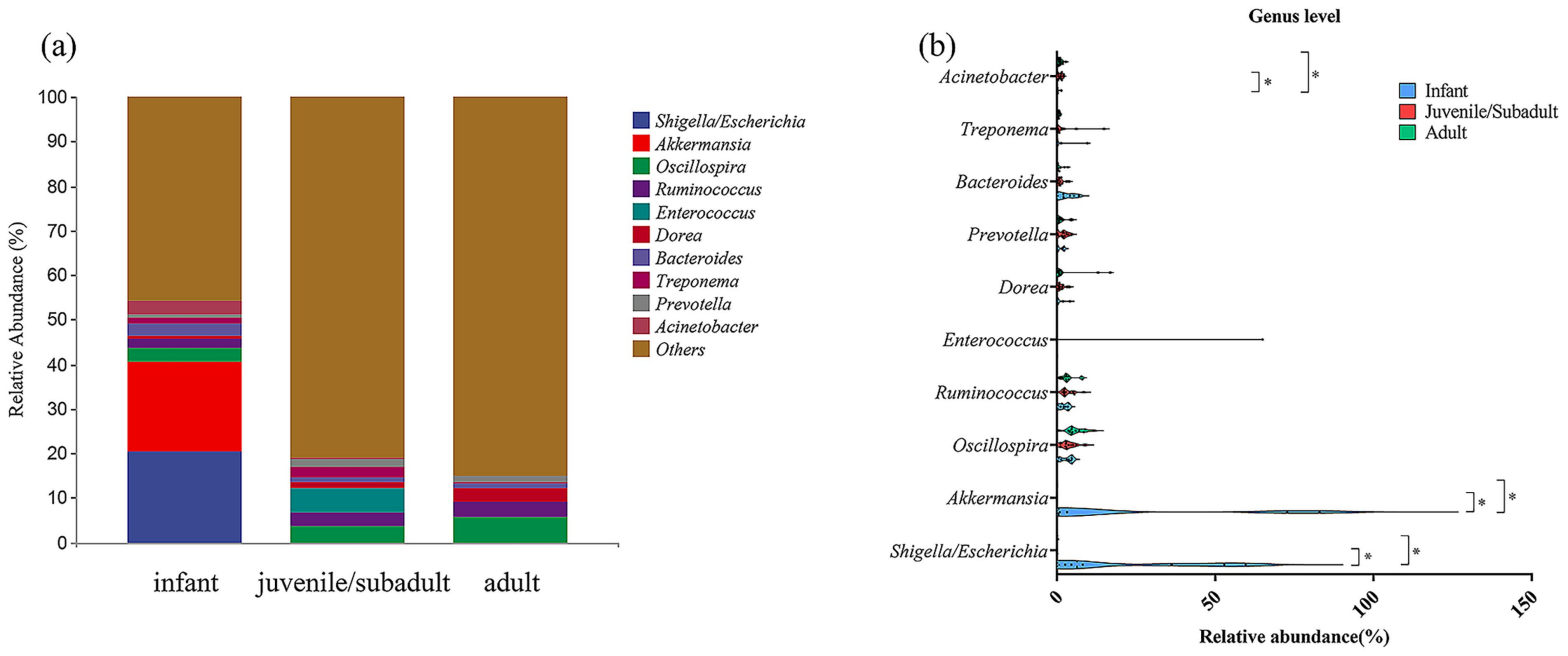
Relative abundance of gut bacteria at Genus level in captive *R. roxellana* at Nanjing Hongshan Forest Zoo, China, March 2022. **(a)** In each age-group; **(B)** comparison of the relative abundance at Genus level in different age classes. The violin plot range indicates the density of the distribution of the data. The straight line represents the median and dots indicate the individual datapoints. * indicates *p* < 0.05, *** indicates *p* < 0.001.

At the genus level, *Shigella/Escherichia* (20.51%), *Akkermansia* (20.00%), and *Oscillospira* (2.90%) were the three genera with the highest abundance in the infant group (Figure 5a). In contrast, the dominant bacterial genera in the juvenile/subadult group were *Oscillospira* (3.51%), *Enterococcus* (5.41%), and *Ruminococcus* (3.28%), while in the adult group, they were *Oscillospira* (5.69%), *Ruminococcus* (3.53%), and *Dorea* (3.05%).

Genera with high relative abundance in the infant group, such as *Shigella/Escherichia* and *Akkermansia*, exhibited a significant decrease in relative abundance during the non-infantile period, respectively (Kruskal-Wallis test, H = 11.64, *p* = 0.0030, df = 2), (Kruskal-Wallis test, H = 9.866, *p* = 0.0072, df = 2) (Figure 5b; Supplementary Table S3). These results suggest that the captive *R. roxellana* population exhibited multiple differences in gut microbiota composition across different age groups.

**Figure 6:**
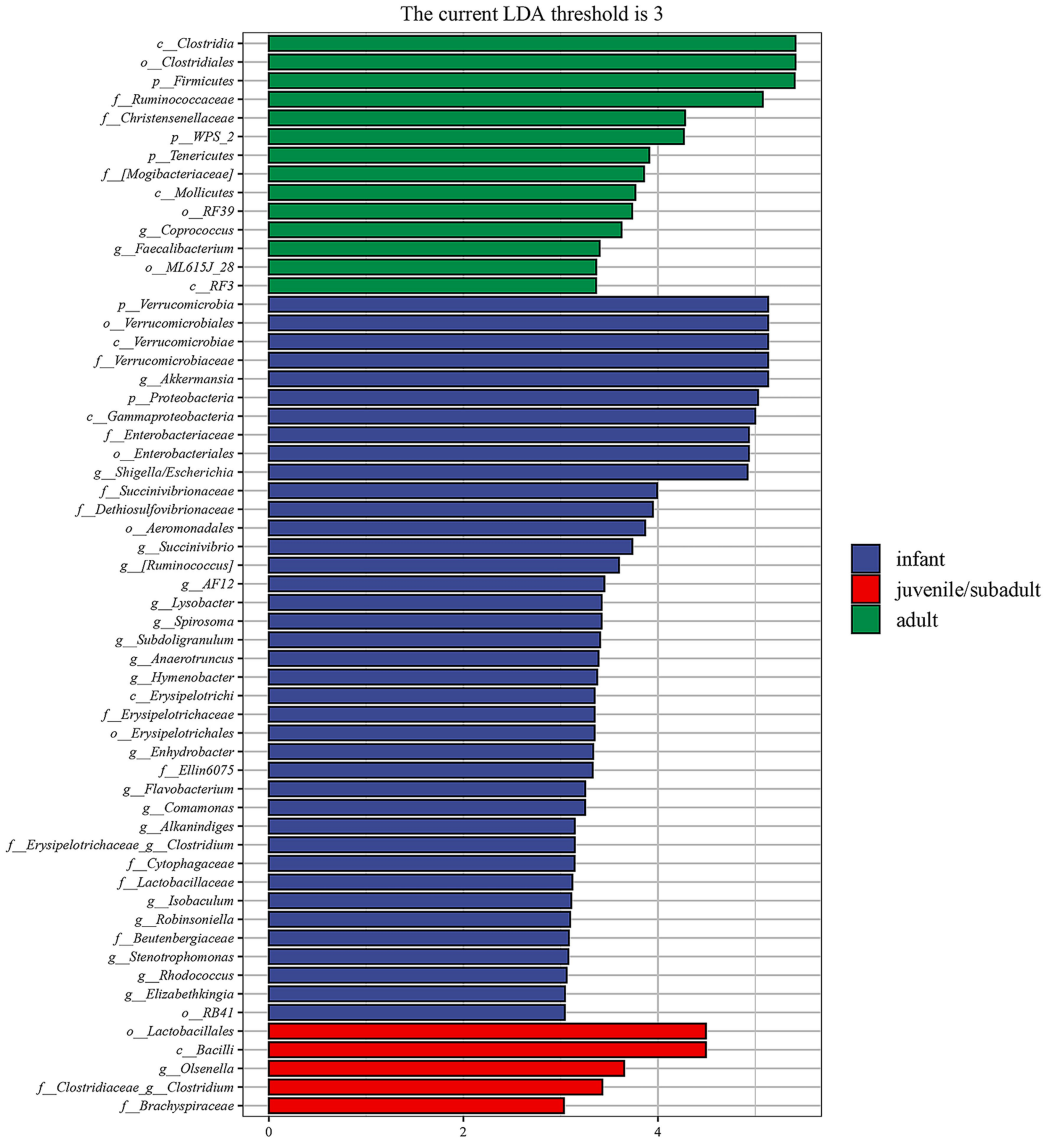
Linear discriminant analysis effect size (LEfSe) analysis of fecal microbiome of *R. roxellana* shows the significantly differential taxa (3-fold, *p <* 0.05) among infant, juvenile/subadult and adult groups.

The differential taxa among three age groups of *R. roxellana* were analyzed using the linear discriminant analysis effect size (LEfSe) method (28). The results depicted in Figure 6 indicate the enriched intestinal microbiota taxa in each group, which exhibited a greater than 3-fold change and a *p*-value of less than 0.05 according to Kruskal-Wallis test. To summarize, the intestinal microbiota of infant *R. roxellana* was significantly enriched with Proteobacteria and Verrucomicrobia at the phylum level, *Shigella/Escherichia* and *Akkermansia* at the genus level. In contrast, the intestinal microbiota of the adult group was significantly enriched with Firmicutes, WPS-2, Tenericutes at the phylum level, and *Coprococcus*, *Faecalibacterium* at the genus level. The intestinal microbiota of juvenile/subadult *R. roxellana* was notably enriched in *the genus Olsenella*. Overall, the intestinal microbiota of the three age groups of *R. roxellana* in captivity displayed significantly different taxa.

### 3.5 Gene function prediction

**Figure 7:**
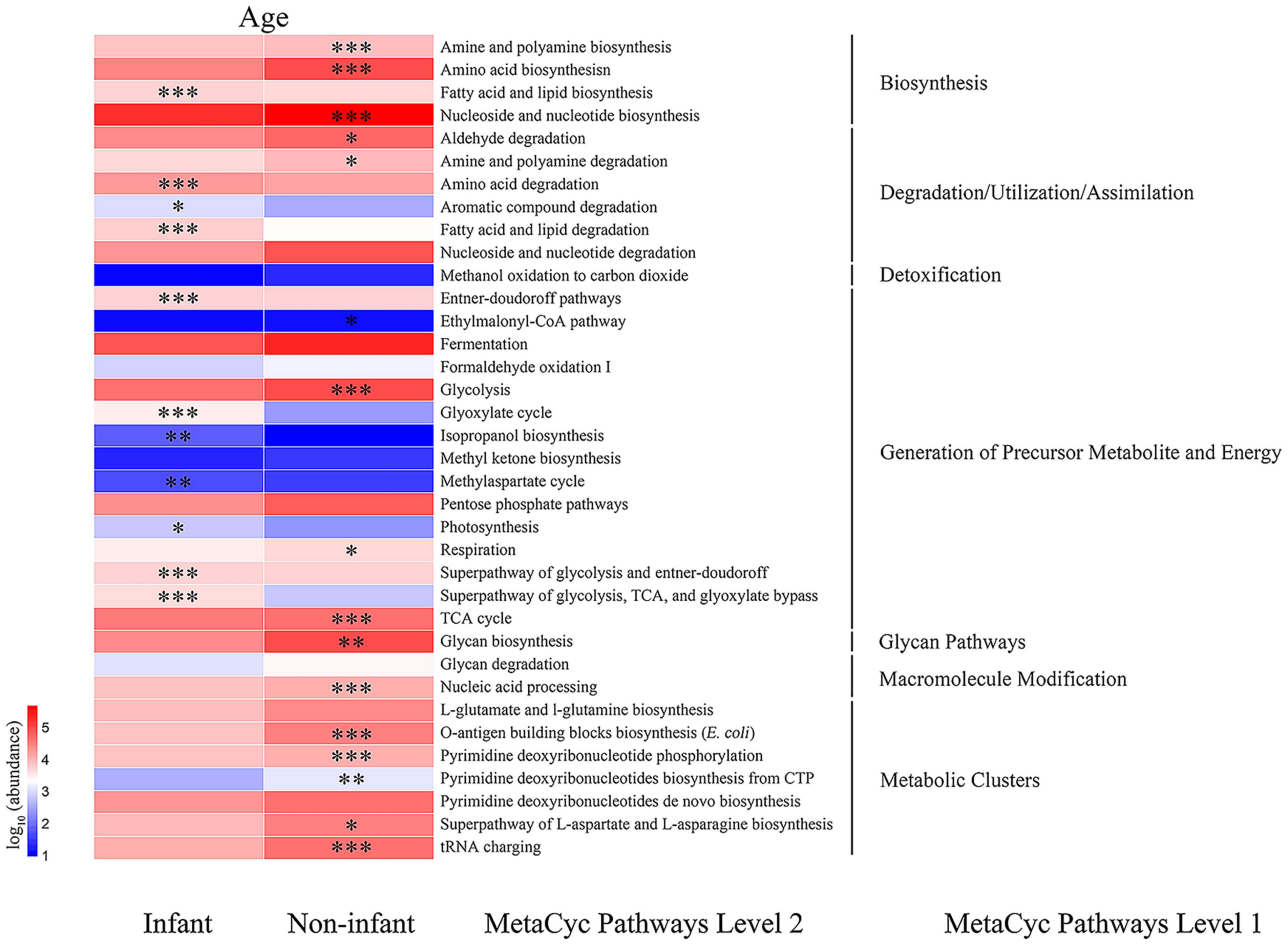
Heatmap of genes with the predicted metabolic function. * indicates *p* < 0.05, *** indicates *p* < 0.001.

The PICRUSt2 method was utilized to predict potential gut microbial gene metabolic functions in *R. roxellana*, drawing from the MetaCyc database. As illustrated in Figure 7, among the predicted first-order metabolic functional pathways, the abundance of genes involved in biosynthesis, macromolecular modification, and metabolic cluster pathways was significantly lower in infant *R. roxellana* compared to non-infant individuals. In contrast, the number of genes involved in degradation, utilization, assimilation, generation of precursors, and energy pathways was significantly higher in infant *R. roxellana* than in non-infant individuals.

Among the predicted second-order metabolic functional pathways, the abundance of multiple genes was significantly higher in the non-infant samples than in infants, including those involved in the biosynthesis of amines, polyamines, amino acids, and others. Genes that were significantly more abundant in the intestines of infants compared to the non-infant group encompassed those responsible for fatty acid and lipid biosynthesis, amino acid degradation, aromatic compound degradation, degradation during fatty acid and lipid degradation/utilization/assimilation, the Entner-Doudoroff pathway, the biosynthetic cycle of isopropanol and methyl aspartate, the glycolytic pathway, and the TCA pathway. It is evident that there are notable differences in the gene functions of intestinal bacteria between infant and non-infant *R. roxellana*.

### 3.6 Identification of cultivable bacteria from the fecal samples

After the bacterial isolation and identification, several culturable bacteria were obtained from the feces of this *R. roxellana* population (Supplementary Table S4). There were nine potential pathogenic bacteria listed in Table 1 and we found that *Enterococcus faecalis* and *Escherichia coli* were isolated from the feces of all age groups, *Aeromonas hydrophila* was obtained from both of infant and adult *R. roxellana*, while *Escherichia fergusonii* was isolated from infant and Juvenile/subadult *R. roxellana* and *Streptococcus gallolyticus* was isolated from non-infant *R. roxellana. Clostridium perfringens, Staphylococcus aureus, Shigella flexneri,* and *Streptococcus viridans* were exclusively isolated from the infant fecal samples. A strain of *Streptococcus agalactiae* was first reported to have been isolated from the feces of a healthy adult *R. roxellana*, and its biological characteristics have already been published (34).

**Table 1:** Culturable opportunistic bacteria isolated from the feces of this captive *R. roxellana* population.

Culture results	Infant	Groups Juvenile/subadult	Adult
*Enterococcus faecalis*	√	√	√
*Escherichia coli*	√	√	√
*Aeromonas hydrophila*	√	–	√
*Escherichia fergusonii*	√	√	
*Streptococcus gallolyticus*	–	√	√
*Clostridium perfringens*	√	–	–
*Staphylococcus aureus*	√	–	–
*Shigella flexneri*	√	–	–
*Streptococci viridans*	√	–	–
*Streptococcus agalactiae*	–	–	√

Ps: The “√” represents that we have isolated the strain. The “–” represents we have not isolated the strain.

## 4 Discussion

It has been observed that the diversity of gut microorganisms is not only related to feeding habits but also to the host’s age (35–37). Among non-human primates, studies on the development of gut microbiota have focused on species such as *Rhesus macaques*, *Rhinopithecus roxellana hubeiensis*, and *Callithrix jacchus* (38–40). Although research on the gut microbiota of *R. roxellana* has been conducted, including analyses of the gut microbiota at various ages and comparisons between captive and wild breeding environments (41, 42), these studies are not yet sufficient to fully describe the characteristics of the gut microbiota in infant *R. roxellana*. In this study, we initially investigated the changes in the gut microbiomes of *R. roxellana* during infancy and non-infancy using 16S rRNA gene sequencing and bacterial culture techniques. These findings offer a better understanding of the gut microbiota characteristics of *R. roxellana* during infancy and elucidate the development of their intestinal microbiota in response to dietary changes throughout development.

In this study, we found that the gut microbiota diversity of *R. roxellana* was remarkably lower in infants than in non-infants, while the differences in microbiota diversity between juvenile/subadult and adult stages were not significant. *R. roxellana* primarily feed on breast milk during infancy, with little difference in feeding habits between juveniles/subadults and adults in captive conditions. The diversity of the intestinal microbiota in captive *R. roxellana* could likely be attributed to their exposure to a wide variety of foods as they age. As the captive population of *R. roxellana* developed, their staple diet shifted from breast milk to one based on leaves. We observed that the composition and dominant microbiota in *R. roxellana* also underwent changes throughout their development. At the phylum level, Firmicutes and Bacteroidetes were the dominant groups in this captive population across all ages, a finding that is consistent with earlier reports (35–37, 43). We discovered that Verrucomicrobia were uniquely predominant in the gut of infant *R. roxellana*. Verrucomicrobia belongs to the Planctomycetes–Verrucomicrobia–Chlamydiae superphylum, which encompasses Chlamydia and Planctomycetes, both of which are notable for their absence of peptidoglycan (44). In recent years, research on Verrucomicrobia within the human gut microbiome has garnered significant interest, particularly regarding the close association between *Akkermansia muciniphila* and human health. *Akkermansia muciniphila*, a member of the phylum Verrucomicrobia, *class Verrucomicrobiae*, and family *Akkermaniaceae,* has been identified as playing a crucial role in promoting metabolic health and enhancing immune function (45). At the family level, *Akkermansia* was also found to be highly abundant in the intestines of infants. Unfortunately, we cannot definitively classify *Akkermansia* at the family level based on current 16S rRNA sequence data, as all *Akkermansia* species exhibit a very high sequence similarity of the full-length 16S rRNA gene (>98%) (46).

We observed that the intestinal microbiota of infant *R. roxellana* was also significantly enriched with Proteobacteria. Proteobacteria play a crucial role in the gut microbiota of humans and animals, being closely related to the balance of the gut microbiota, overall health, metabolic functions, immune regulation, and pathogenicity (47). In the human gut, the increased abundance of Proteobacteria can reflect an unstable gut microbiota structure (48) when non-pathological changes during neonatal period (49), gastric bypass surgery (50), metabolic disorders (51) and intestinal inflammation (52) could cause this instability. Many human pathogens belong to the phylum Proteobacteria: *Brucella*, *Bordetella*, *Escherichia*, *Shigella*, *Salmonella*, and so on (47). Recent studies suggest that the phylum Proteobacteria may serve as a microbial marker for gut diseases (53, 54). Our LEfSe analysis also revealed that one of the differential microbiota in infant *R. roxellana* was *Shigella/Escherichia*. Regrettably, the 16S rRNA gene sequenced in our study cannot effectively distinguish between *Shigella and Escherichia,* DEVANGA *et. al* also verified this phenomenon (55). Both *Shigella* and *Escherichia* are commensal bacteria in primates intestinal, but certain strains of these bacteria can cause diarrhea and death in severe cases (56). The abundance changes of *Shigella/Escherichia* in the intestines of *R. roxellana* with ages may reflect the evolutionary colonization of Proteobacteria, but it also suggested that infant *R. roxellana* exhibited relatively unstable gut microbiota structures and faced more challenges from gut diseases than non-infants. Further studies should be conducted based on correlation between *Shigella/Escherichia* and infanct digestive-related diseases in *R. roxellana*.

We reported for the first time that a strain of *Streptococcus agalactiae,* named CFFB, was isolated from a healthy adult *R. roxellana*. Our subsequent phylogenetic and genome comparison analyses revealed that CFFB shared a similar serotype (III), genotype (ST19), pilus types (PI-1 + PI-2a), and CRISPR spacers with human clinical strains from China, although the virulence of CFFB for mice was weaker than that of human strain *S. agalactiae* ATCC BAA-611 (34). These findings suggest that CFFB isolated from *R. roxellana* may originate from human *S. agalactiae* and possesses potential zoonotic risks. It also reminds us that we must continuously monitor the pathogenic microorganisms carried by captive wild animals in close contact with humans to reduce the risk of zoonotic diseases.

## 5 Conclusion

In this study, we found that the diversity of the intestinal microbiota during infancy was lower than that in non-infancy within this captive population of *R. roxellana*. Both the species and relative abundance of the intestinal microbiota of *R. roxellana* during infancy differed from those in non-infancy. Meanwhile, the intestines of infant *R. roxellana* faced more challenges from potentially pathogenic bacteria than those of non-infants. With age changes and dietary transitions, the intestinal microbiota began to evolve toward that of adulthood. The experimental results suggest that we should carefully prepare clean food for the captive weaning infant *R. roxellana*, implement additional measures to maintain the stability of the intestinal microbiota, and prevent the proliferation of potentially pathogenic bacteria during the dietary transition period. Our research provides a basis for further understanding of the changes in the characteristics of the intestinal microbiota of *R. roxellana* during development and contribute to improve the survival of infant *R. roxellana* in captive breeding.

## Data Availability

The original contributions presented in the study are publicly available. The 32 raw 16S rRNA sequencing datasets generated in this study have been deposited in the National Center for Biotechnology Information (NCBI) Sequence Read Archive (SRA) repository. This data can be found here: https://www.ncbi.nlm.nih.gov/sra/?term=PRJNA1337937 (Project accession number: PRJNA1337937).
