# Supplementary material for: The variation of gut microbiota in captive Sichuan golden snub-nosed monkeys (Rhinopithecus roxellana) from infancy to adulthood
Source: Front Vet Sci. 2025 Oct 28;12:1683047. doi: 10.3389/fvets.2025.1683047 (PMC12604355; doi:10.3389/fvets.2025.1683047)
Supplement: Supplementary file 1 [file Data_Sheet_1.docx]

Supplementary Material

# Supplementary Figures and Tables

## Supplementary Figures


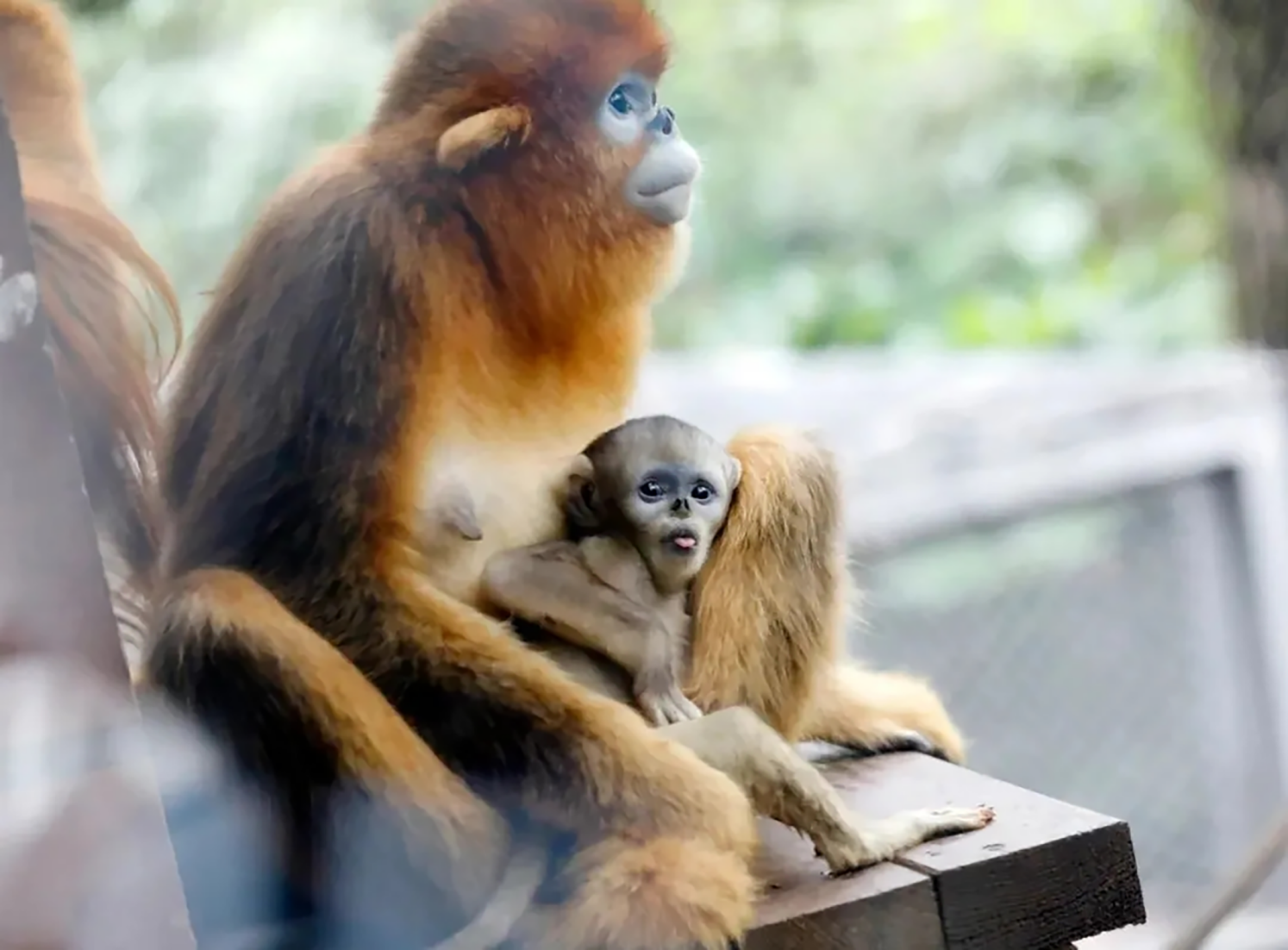


**Supplementary Figure S1.** 2 month old breastfed infant *R. roxellana* in its mother embrace

**Supplementary Figure S2.** The relative abundance for 32 fecal samples from *R. roxellanae* (a). Rarefaction curves obtained by plotting the number of ASVs with the number of reads (b-f)

## Supplementary Tables

**Supplementary Table S1.** The detailed information of samples

| **Group** | **No.** | **Date of birth** | **Age at sampling** | **Gender** | **Accession** |
| --- | --- | --- | --- | --- | --- |
| **Infant** | \| 1 \| \| --- \| \| 2 \| \| 3 \| \| 4 \| \| 5 \| \| 6 \| \| 7 \| \| 8 \| | \| 2022.04.05 \| \| --- \| \| 2022.03.27 \| \| 2022.03.28 \| \| 2022.04.10 \| \| 2022.04.13 \| \| 2022.04.01 \| \| 2022.04.20 \| \| 2022.03.21 \| | \| 14 days \| \| --- \| \| 15 days \| \| 16 days \| \| 14 days \| \| 14 days \| \| 16 days \| \| 17 days \| \| 18 days \| | \| Female \| \| --- \| \| Female \| \| Female \| \| Female \| \| Male \| \| Male \| \| Male \| \| Male \| | \| SRR35742062 \| \| --- \| \| SRR35742061 \| \| SRR35742045 \| \| SRR35742034 \| \| SRR35742031 \| \| SRR35742059 \| \| SRR35742058 \| \| SRR35742057 \| |
| **Juvenile/**  **subadult** | \| 1 \| \| --- \| \| 2 \| \| 3 \| \| 4 \| \| 5 \| \| 6 \| \| 7 \| \| 8 \| \| 9 \| \| 10 \| \| 11 \| \| 12 \| | \| 2017.03.26 \| \| --- \| \| 2017.03.28 \| \| 2017.05.04 \| \| 2018.04.28 \| \| 2018.05.05 \| \| 2019.04.01 \| \| 2019.04.29 \| \| 2019.05.08 \| \| 2019.05.02 \| \| 2020.04.10 \| \| 2020.03.31 \| \| 2020.04.05 \| | \| 5 years \| \| --- \| \| 5 years \| \| 5 years \| \| 4 years \| \| 4 years \| \| 3 years \| \| 3 years \| \| 3 years \| \| 3 years \| \| 2 years \| \| 2 years \| \| 2 years \| | \| Female \| \| --- \| \| Male \| \| Female \| \| Female \| \| Female \| \| Female \| \| Male \| \| Female \| \| Male \| \| Male \| \| Male \| \| Female \| | \| SRR35742056 \| \| --- \| \| SRR35742055 \| \| SRR35742060 \| \| SRR35742054 \| \| SRR35742053 \| \| SRR35742052 \| \| SRR35742051 \| \| SRR35742050 \| \| SRR35742049 \| \| SRR35742048 \| \| SRR35742047 \| \| SRR35742046 \| |
| **Adult** | \| 1 \| \| --- \| \| 2 \| \| 3 \| \| 4 \| \| 5 \| \| 6 \| \| 7 \| \| 8 \| \| 9 \| \| 10 \| \| 11 \| \| 12 \| | \| 2008.04.26 \| \| --- \| \| 2008.04.15 \| \| 2009.04.23 \| \| 2009.03.27 \| \| 2011.05.28 \| \| 2012.04.14 \| \| 2015.04.21 \| \| 2015.04.15 \| \| 2014.03.27 \| \| 2014.03.28 \| \| 2014.04.14 \| \| 2014.04.17 \| | \| 14 years \| \| --- \| \| 14 years \| \| 13 years \| \| 13 years \| \| 11 years \| \| 10 years \| \| 8 years \| \| 8 years \| \| 7 years \| \| 7 years \| \| 7 years \| \| 7 years \| | \| Female \| \| --- \| \| Male \| \| Female \| \| Female \| \| Male \| \| Female \| \| Female \| \| Male \| \| Male \| \| Male \| \| Female \| \| Female \| | \| SRR35742044 \| \| --- \| \| SRR35742043 \| \| SRR35742042 \| \| SRR35742041 \| \| SRR35742040 \| \| SRR35742039 \| \| SRR35742038 \| \| SRR35742037 \| \| SRR35742036 \| \| SRR35742035 \| \| SRR35742033 \| \| SRR35742032 \| |

**Supplementary Table S2.** Results of Kruskal-Wallis test comparing the relative abundance of bacteria at phyla level among three age classes

| Phylum | H | p | df |
| --- | --- | --- | --- |
| Firmicutes | 16.76 | 0.0002** | 2 |
| Bacteroidetes | 2.949 | 0.2289 | 2 |
| Proteobacteria | 14.82 | 0.0006** | 2 |
| Verrucomicrobia | 8.956 | 0.0114* | 2 |
| WPS-2 | 7.535 | 0.0231* | 2 |
| Spirochaetes | 2.257 | 0.3205 | 2 |
| T enericutes | 13.01 | 0.0015* | 2 |
| Actinobacteria | 1.189 | 0.5517 | 2 |
| Cyanobacteria | 4.343 | 0.1089 | 2 |
| Fibrobacteres | 0.5844 | 0.7466 | 2 |

Note: * *p* < 0.05, ***p* < 0.01.

**Supplementary Table S3.** Results of Kruskal-Wallis tests comparing the relative abundance of bacteria at genus level among different age groups

| Genera | H | p | df |
| --- | --- | --- | --- |
| *Shigella/Escherichia* | 11.64 | 0.003** | 2 |
| *Akkermansia* | 9.866 | 0.0072** | 2 |
| *Oscillospira* | 5.288 | 0.0711 | 2 |
| *Ruminococcus* | 1.894 | 0.3879 | 2 |
| *Ruminococcus* | 4.906 | 0.0861 | 2 |
| *Dorea* | 3.108 | 0.2114 | 2 |
| *Prevotella* | 3.389 | 0.1837 | 2 |
| *Bacteroides* | 2.282 | 0.3195 | 2 |
| *Treponema* | 2.238 | 0.3267 | 2 |
| *Coprococcus* | 10.47 | 0.0053** | 2 |

Note: * *p* < 0.05, ***p* < 0.01.


**Supplementary Table S3.** The bacteria isolated from from the faeces of this captive *R. roxellana* population

| **Group** | **Non-pathogenic strains** | **Potential Pathogenic strains** |
| --- | --- | --- |
| **Infant** | *Bacteroides vulgatus,*  *Citrobacter werkmanii,*  *Lactobacillus animali* | *Aeromonas hydrophila*, *Cronobacter sakazakii* ,*Clostridium perfringens*, *Escherichia fergusonii, Escherichia coli, Escherichia* faecalis, *Erysipelatoclostridium ramosum,*  *Morganella morganii, Klebsiella oxytoca,*  Paraclostridium bifermentans, *Staphylococcus aureus, Staphylococcus pasteuri*, *Shigella flexneri*, *Shigella, Streptococci Viridans* |
| **Juvenile/**  **subadult** | *Bacteroides vulgatus,*  *Bacteroides thetaiotaomicron,*  *Bacillus firmus,*  *Clostridium neonatale,*  *Erysipelatoclostridium ramosum,*  *Lactobacillus mucosae,*  *Lactobacillus fermentum,*  *Phocaeicola sartorii,*  *Staphylococcus equorum* | *Acinetobacter lwoffii*, *Bacillus cereus,*  *Escherichia coli*, *Escherichia* faecalis,  *Escherichia fergusonii*,  *Kocuria carniphila*, *Streptococcus gallolyticus* |
| **Adult** | *Aerococcus sarguinicola,*  *Aerococcus urinaehominis,*  *Bacillus albus,*  *Bacteroides sartorii,*  *Bacteroides xylanisolvens,*  *Bacteroides dorei,*  *Bacteroides uniformis,*  *Lactobacillus mucosas,*  *Bacteroides vulgatus,*  *Clostridium neonatale,*  *Lactobacillus fermentum,*  *Paraclostridium benzoelyticum,*  *Phocaeicola sartorii ,*  *Parabacteroides distasonis,*  *Peribacillus endoradicis,* | *Aeromonas hydrophila, Escherichia coli, Escherichia faecalis*, *Enterococcus faecium*,  Paraclostridium bifermentans, *Pantoea agglomerans, Streptococcus gallolyticus*,  *Streptococcus agalactiae* |
